# Supplementary material for: Cloud BioLinux: pre-configured and on-demand bioinformatics computing for the genomics community
Source: BMC Bioinformatics. 2012 Mar 19;13:42. doi: 10.1186/1471-2105-13-42 (PMC3372431; doi:10.1186/1471-2105-13-42)
Supplement: Additional file 1 — Supplementary 1 Cloud BioLinux software documentation in the form of a mini, self-contained website. Users need to download and uncompress the .zip file, and open through a web browser the "index.html" file available on the main directory. (ZIP 1823 kb). [file 1471-2105-13-42-S1.ZIP › Cloud-BioLinux-Package-Documentation/Nucleic.html]

Bio-Linux Software Documentation Pages

Back to search form

### Nucleic

|  |  |
| --- | --- |
| Category | Applications |
| Nucleic |  |
| SubCategory | Applications |
| 2d\_structure | arb    einverted    garnier    helixturnhelix    hmoment    newcoils    pepcoil    pepnet    pepwheel    tmap |
| Annotation | act    artemis    partigene    qiime    rdp\_classifier |
| Assembly | bwa    cap3    consed    gcphrap    mira    partigene    phrap    ssake    velvet |
| Base\_calling | phred    pregap4 |
| Codon\_usage | cai    chips    codcmp    codon-usage    cusp    syco |
| Composition | backtranambig    backtranseq    banana    btwisted    chaos    charge    checktrans    complex    compseq    compseq    dan    density    emowse    freak    freak    get-len    giep    iep    isochore    mwcontam    mwfilter    octanol    pepinfo    pepstats    pepwindowall    sirna    wordcount    wordcount |
| Cpg\_islands | cpgplot    cpgreport    geecee    newcpgreport    newcpgseek |
| Est | cd-hit-est    estscan    estwise    estwisedb    genewise    genewisedb    partigene    prot4est    sibsim4    sim4    trace2dbest |
| Gene\_finding | act    anomaly    artemis    build-icm    check    codon-usage    compare-lists    estscan    estwise    estwisedb    extract    generate    genewise    genewisedb    get-putative    getorf    glimmer2    long-orfs    marscan    plotorf    prot4est    run-glimmer2    showorf    sibsim4    sim4    sixpack    syco    tcode    wobble |
| Graphical | act    artemis |
| Masking | dust    mdust |
| Motifs | antigenic    digest    dreg    epestfind    fuzznuc    fuzzpro    fuzztran    fuzztran    glam2    helixturnhelix    marscan    ocount    oddcomp    patmatdb    patmatmotifs    pepcoil    sigcleave |
| Mutation | msbar    msbar    pregap4    shuffleseq    shuffleseq |
| Nextgen | bwa    caftools    consed    denoiser    maq    mira    mira-3rd-party    qiime    roche2gap    samtools    ssake    tqs    velvet |
| Primers | eprimer3    OligoArray    prima    primer3    primers    primersearch    stssearch |
| Profiles | jprofilegrid    profit    profit    prophecy    prophecy    prophet    prophet |
| Repeats | cross\_match    dust    dustmasker    einverted    equicktandem    etandem    mdust    palindrome    windowmasker |
| Restriction | recoder    redata    remap    restdist    restml    restover    restrict    showseq    silent |
| Transcription | jaspscan    tfscan |
| Translation | 6ft    backtranambig    backtranseq    coderet    plotorf    prettyseq    remap    showorf    showseq    sixpack    tfastx    tfasty    transeq |
